# Systematic Screening for Occupational Exposures in Lung Cancer Patients: A Prospective French Cohort

**DOI:** 10.3390/ijerph15010065

**Published:** 2018-01-04

**Authors:** Olivia Pérol, Barbara Charbotel, Lionel Perrier, Sandrine Bonnand, Elodie Belladame, Virginie Avrillon, Paul Rebattu, Frédéric Gomez, Géraldine Lauridant, Maurice Pérol, Beatrice Fervers

**Affiliations:** 1 Département Cancer et Environnement, Centre Léon Bérard, 69373 Lyon CEDEX 08, France; elodie.belladame@lyon.unicancer.fr (E.B.); beatrice.fervers@lyon.unicancer.fr (B.F.); 2 Université Lyon 1, UMRESTTE (Unité Mixte IFSTTAR/UCBL), 69373 Lyon CEDEX 03, France; barbara.charbotel-coing-boyat@univ-lyon1.fr; 3 Hospices Civils de Lyon, Service des Maladies Professionnelles, Centre Hospitalier Lyon Sud, 69495 Pierre Bénite, France; 4 Université Lyon, Centre Léon Bérard, GATE L-SE UMR 5824, F-69008 Lyon, France; lionel.perrier@lyon.unicancer.fr; 5 Département Interdisciplinaire de soins de Support du Patient en Oncologie, Service Social, Centre Léon Bérard, 69373 Lyon CEDEX 08, France; sandrine.bonnand@hopital-fourviere.fr; 6 Département d’Oncologie Médicale, Centre Léon Bérard, 69373 Lyon CEDEX 08, France; virginie.avrillon@lyon.unicancer.fr (V.A.); paul.rebattu@lyon.unicancer.fr (P.R.); maurice.perol@lyon.unicancer.fr (M.P.); 7 Département d’Information Médicale, Centre Léon Bérard, 69373 Lyon CEDEX 08, France; frederic.gomez@lyon.unicancer.fr; 8 Département d’Oncologie Médicale, Centre Oscar Lambret, 59000 Lille, France; g-lauridant@o-lambret.fr; 9 Centre de Recherche en Cancérologie de Lyon, UMR INSERM 1052-CNRS 5286, F-69008 Lyon, France

**Keywords:** lung cancer, occupational exposures, systematic self-administered questionnaire, cost analysis, social deprivation

## Abstract

Occupational lung cancers are under-reported and under-compensated worldwide. We assessed systematic screening for occupational exposure to carcinogens combining a self-administered questionnaire and an occupational consultation to improve the detection of occupational lung cancers and their compensation. Social deprivation and the costs of this investigation were estimated. Patients with lung cancer received a self-administered questionnaire to collect their job history, potential exposure to carcinogens and deprivation. A physician assessed the questionnaire and recommended an occupational consultation if necessary. During the consultation, a physician assessed if the lung cancer was work-related and, if it was, delivered a medical certificate to claim for compensation. Over 18 months, 440 patients received the self-administered questionnaire: 234 returned a completed questionnaire and a consultation was required for 120 patients. Compensation was judged possible for 41 patients. Among the 35 medical certificates delivered, 19 patients received compensation. Nearly half the patients (46%) were assessed as socially deprived and these patients took significantly longer to return the questionnaire compared with those who were not deprived. The mean cost of the process was €62.65 per patient. Our results showed a systematic self-administered questionnaire can be used to identify patients potentially exposed to carcinogens and to improve compensation.

## 1. Introduction

Lung cancer is the most common cancer (12.9% of all cancers) with 1.8 million new cases diagnosed worldwide annually [1]. It is the most common cause of cancer death worldwide with 1.5 million deaths reported in 2012. Tobacco consumption accounts for 80–85% of lung cancers [2,3] and secondhand smoking is also an established risk factor [4,5]. Genetic susceptibility [6], air pollution [7] and occupational exposure [6,8,9] also play a significant role in lung cancer etiology. The International Agency for Research on Cancer (IARC) has identified several agents present in the occupational sector that are associated with lung cancer [10]. Synergistic carcinogenic effects of tobacco smoke with occupational exposure have also been reported [11]. Lung cancer patients have a lower socio-economic status compared with the general population [12] related to a higher prevalence of risk factors such as smoking and exposure to occupational lung carcinogens [4,13,14].

The population attributable fraction (PAF) of lung cancer deaths due to occupational carcinogens has been estimated at between 8% and 24% worldwide [15,16,17,18,19]. The range is wide mainly because the studies did not consider the same occupational exposure and the number of exposed workers varied over time and by country. Overall, lung cancer accounts for more than half of all occupational cancers [17,19].

Occupational cancers are largely under-reported and under-compensated; in France, only 2.3% of lung cancers (including 2.1% related to asbestos) have been compensated in 2014 [20]. Under-reporting can be explained by several factors: there is limited knowledge about occupational exposure among physicians [21] and generally, there is a lack of interest and time to collect occupational history [22]. The stigma associated with smoking [22,23], the long latency and changes in exposure patterns over time are barriers both at physician and patient levels. Patients in the most frequently-exposed socio-professional categories often overlook job-related exposure [24]. Moreover, patients with a low socio-economic status experience greater difficulty with administrative processes [22,25]. The poor prognosis of lung cancers, with a five-year survival below 20% [26], also contributes to under-reporting [24] and under compensation.

The medical and carcinogen exposure conditions defining occupational lung cancer and the compensation processes are heterogeneous among countries [27]. Several studies assessing interventions in physician practices reported that they did not substantially improve reporting of occupational diseases [21,22,28,29,30].

The present study assessed systematic screening of occupational exposure, combining a self-administered questionnaire (SAQ) and a specialized occupational cancer consultation to improve the detection of occupational lung cancers and their compensation as occupational disease. Social deprivation (using a specific questionnaire) and the costs of systematically investigating occupational exposure and of hospitalization for occupational lung cancer were also assessed.

## 2. Materials and Methods

The study was declared to the Medical Committee for Protection of Personal Data (No. 1737645). The study was conducted in accordance with the Declaration of Helsinki, and the protocol was approved by the Ethics Committee of the Regional Comprehensive Cancer Center Léon Bérard (CLB), Lyon, France.

### 2.1. Study Population

Patients (male and female with no age limitation) treated at the CLB for lung cancer between March 2014 and September 2015 who spoke French were eligible. Patients managed elsewhere, or seen in the CLB for radiotherapy, diagnostic procedures, or medical second opinion were not eligible.

### 2.2. Self-Administered Questionnaire for Occupational Exposure Screening

Eligible patients, identified through the CLB weekly multidisciplinary lung cancer board were sent an information letter, the SAQ and a prepaid return envelope. The SAQ, which had been validated in a sub-sample of 89 patients from the study [31], collected information about the patients’ education level, job history (job-title, start and end dates, employer and sector of activity, and tasks performed) as well as exposure to 25 lung carcinogens during their career. When the patients had not returned the SAQ after three weeks, a research technician called and offered help to complete it. At reception, the SAQ was assessed by a physician to determine if a specialized occupational cancer consultation was required, based on the jobs, tasks and exposure reported by the patient. When no occupational exposure was identified, the patients were sent a personalized letter informing them that their disease was assessed as not work-related.

### 2.3. Individual Social Deprivation

Patients were also asked to complete the Evaluation of Deprivation and Inequalities in Health Examination Centers (EPICES) score (sent with the SAQ), a validated composite index used to measure individual deprivation [32]. The EPICES score, which is composed of 11 binary (yes/no) items covering marital status, health insurance status, economic status, family support and leisure activity, has been shown to be strongly correlated with several health indicators [32]. The score ranges from 0 (no deprivation) to 100 (maximal deprivation) with a threshold at 30.

### 2.4. Occupational Cancer Consultation

During the consultation, the physician collected data on the patient’s job history, exposure to carcinogens, conditions, frequency, duration and level of exposure, means of protection and non-work-related risk factors (i.e., smoking history and non-occupational exposure, in particular to asbestos). In France, there is a list of occupational diseases that specifies the symptoms or pathological lesions required, the type of work known to cause the condition and the time limits for compensation claims [27]. Any disease satisfying these medical, occupational and administrative conditions is systematically assumed to be work-related, without considering any potential non-occupational factors. When the disease is not mentioned on the list or the criteria are not fully met, patients are examined by regional committees for occupational diseases recognition, who usually base their assessment on IARC Group 1 classification and consider non-occupational factors, in particular smoking [33]. In addition, patients who had been exposed to asbestos (occupational-related or not) were given a medical certificate to make a claim to the asbestos victim compensation fund (In French; *Fonds d’Indemnisation des Victimes de l’Amiante*, FIVA (http://www.fiva.fr/)). In the study, if the physician judged the lung cancer to be occupational-related, a medical certificate, required for compensation claims, was delivered. Patients who wanted to claim for compensation were offered help from a social worker at the CLB for the claim process.

### 2.5. Data Collection

In addition to demographic data and data collected through the SAQ, the EPICES score and the occupational cancer consultation, we collected data on clinical and tumor characteristics and smoking history from the patient’s medical record. All consultations were registered in the database of the National Network for Monitoring and Prevention of Occupational Diseases [34].

### 2.6. Cost Assessment

The cost assessment (in 2014 euros) was based on a bottom-up micro-costing approach from the healthcare providers perspective [35]. Three situations were used to estimate costs: the SAQ was returned by the patient and an occupational cancer consultation was required (*Situation 1*); the SAQ was returned by the patient and no consultation was required (*Situation 2*); and the SAQ was not returned by the patient after three phone calls (*Situation 3*). Data on resource consumption between the SAQ administration and the physician’s evaluation during the consultation, and social worker costs, if applicable were also collected. Details are provided in Appendix A. The cost for implementing the systematic investigation of occupational exposure for lung cancers throughout France was then estimated, based on all incident cases. The costs of occupational lung cancer hospitalizations were based on the tariffs corresponding to the *Groupes Homogènes de Malades*, which is the French equivalent of diagnosis related group (DRG). A one-year time horizon from diagnosis was used (see Supplementary Materials for more details).

### 2.7. Statistical Analysis

Data were analyzed using descriptive statistics, *t*-tests and Mann–Whitney tests for comparisons of quantitative data and Chi-2 and Fisher exact tests for qualitative data. All enrolled patients were included in the analysis.

One-way sensitivity analyses were conducted by varying resource consumption and unit cost parameters by ±10% and the results displayed in Tornado diagrams. The uncertainty surrounding costs was assessed by probabilistic sensitivity analyses using a non-parametric bootstrap method: 1000 simulated bootstrap samples were generated by independent draws for *Situations 1*, *2*, and *3*. All statistical analyses were performed using R (version 3) and STATA^®^ (version 14.0 StataCorp. LP; College Station, TX, USA) software.

## 3. Results

Between March 2014 and September 2015, 588 of the 1028 patients who were screened by the weekly CLB multidisciplinary lung cancer board did not meet the inclusion criteria: managed in another hospital (*N* = 340); not lung cancer (*N* = 81); only radiotherapy in the CLB (*N* = 46); already had had an occupational consultation (*N* = 39); no histological confirmation (*N* = 25); benign pathology (*N* = 24); second opinion (*N* = 14); deceased (*N* = 10); severe general health deterioration (*N* = 8); and did not speak French (*N* = 1). The remaining 440 patients (43%) were enrolled.

### 3.1. Patients’ Characteristics

The patients’ characteristics are summarized in Table 1. Most patients (83%) were smokers (current/ex-smokers). Smokers were significantly younger (62.5 years (SD = 10.6)) than non-smokers (65.7 years (SD = 11.5)) (*p* = 0.03). The majority of patients were diagnosed with non-small cell lung cancer (91%) and metastatic disease (55%). No differences were observed between responders and non-responders for gender, age, cancer stage, smoking status and the patient’s clinical situation (Table 2).

### 3.2. Self-Administered Questionnaire

**Figure 1:**
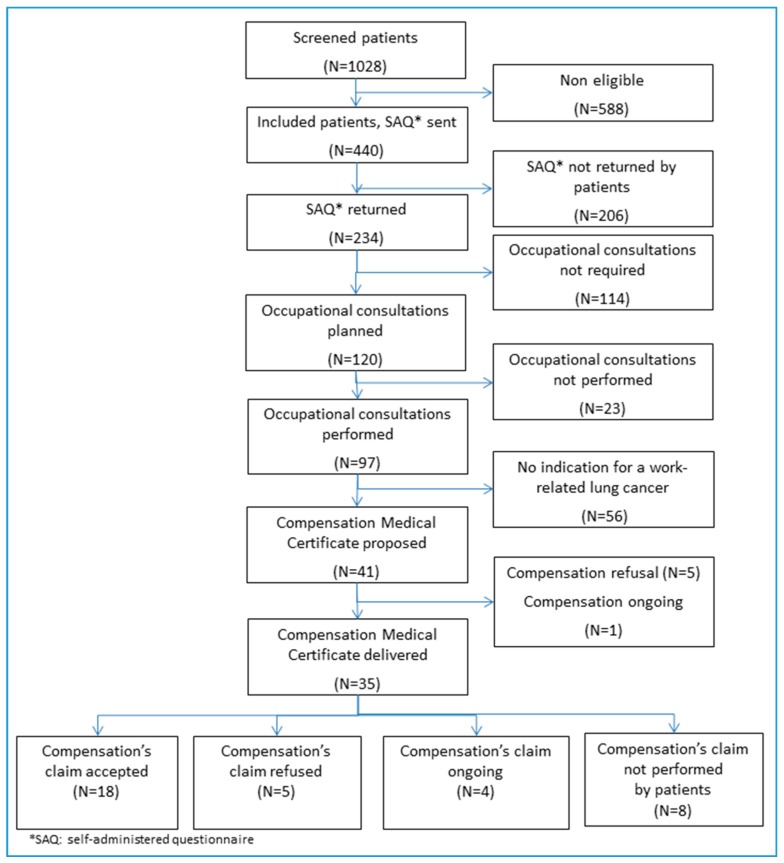
Study flow-chart.

The process is presented in Figure 1. The SAQ was sent to 440 eligible patients and returned by 234 (53%): 129 without reminder; 105 after phone contact. Some patients (4%) completed the SAQ with technician’s help. Among the 206 patients who did not complete the SAQ, 84 patients (19%) declared they did not feel concerned and 32 patients (7%) could not be contacted by phone after three attempts (Table 3). The average delay for returning the SAQ was 47 days. Newly diagnosed patients returned the SAQ more frequently within the first three weeks than those with disease progression (*p* = 0.03).

The responders had an average of 3.4 jobs (range: 0–20) with a mean duration of 7.9 years (SD = 10.6) per job. A quarter of the patients reported having been exposed to at least one carcinogen during their career and 17 patients to more than five carcinogens. The most frequent carcinogens were asbestos (25%), second-hand smoke (25%) and diesel engine exhausts (19%). An occupational cancer consultation was proposed to 120 patients (51%).

### 3.3. Occupational Cancer Consultation

A total of 97 of the 120 invited patients (80%) attended the consultation. The main reasons for non-attendance were death (7%), patient felt unconcerned (6%) or did not want to continue the process (6%). Among the patients who attended the consultation, 59 patients (61%) were considered to have occupational-related lung cancer. The main occupational exposures were asbestos (53%), welding fumes (13%) and diesel engine exhaust (12%). A claim for compensation was judged possible under the French system for 41 patients and the mandatory medical certificate was delivered to 35 patients. Five patients did not want to claim and one patient had already filed a claim. A compensation claim was judged impossible or unlikely to be successful for 18 patients (criteria for compensation not fulfilled and/or presence of an extra-occupational factor (i.e., smoking)) (6%); or the factor was not considered as a carcinogen in the French system (13%). For the remaining 38 patients, lung-cancer was not considered to be work-related, or no relevant occupational exposure was identified. Compensation was awarded to 19 patients (54%) (Table 4). Five claims (17%) were rejected, three are still under assessment and eight patients (23%) did not submit a claim.

### 3.4. Individual Social Deprivation

The mean EPICES score was 28.7. Nearly half the patients (46%) had an EPICES score >30 and were considered to be socially deprived. On average, patients classified as deprived took significantly longer to return the SAQ compared with those who were not (56 days versus 38 days; *p* = 0.002) and were phoned more frequently (*p* = 0.05). The need for an occupational cancer consultation and the right to compensation were not significantly different between deprived and non-deprived patients. Among the patients who did not apply for proposed compensation, 75% were identified as deprived.

### 3.5. Cost Evaluation

**Figure 2:**
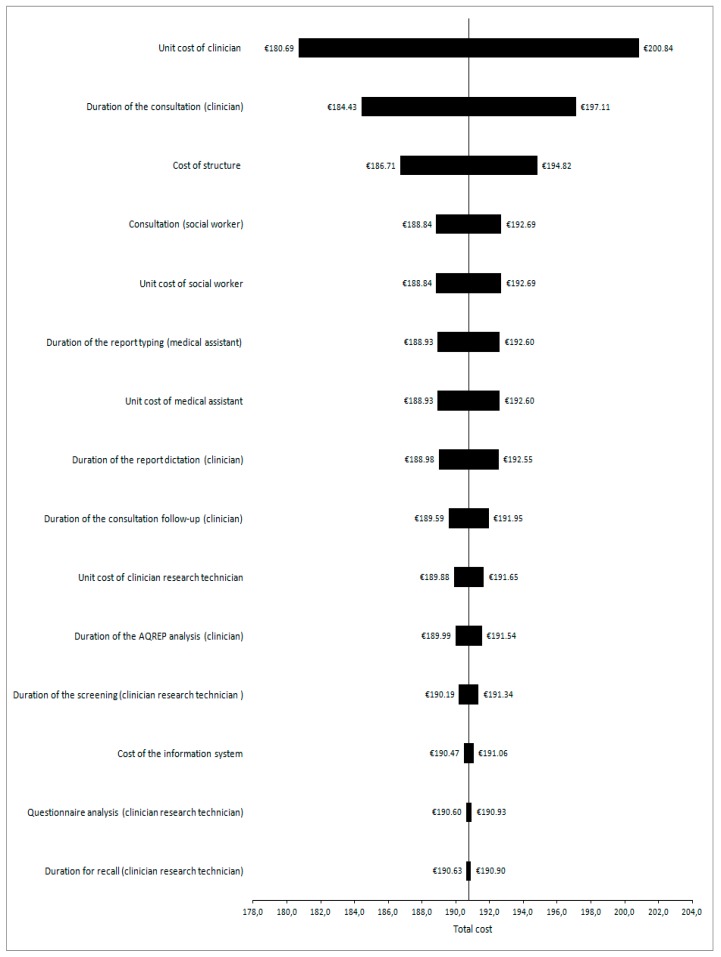
Tornado diagram for *Situation 1* (Self-administered questionnaire (SAQ) returned by the patient and consultation required based on the information provided by the patient in the SAQ).

The mean costs per patient for *Situation 1* (*N* = 77) were €190.77 (SD €96.20; 95% CI €169.9–211.6); for *Situation 2* (*N* = 82) €24.52 (SD €15.3; 95% CI €21.4–27.7); and for *Situation 3* (*N* = 142) €13.69 (SD €3.61; 95% CI €13.1–14.3) (Table 5). Analyses showed that estimated costs were most sensitive to the unit cost of physician in *Situation 1*, a 10% increase (i.e., from €1.51 to €1.67 per minute) increasing the mean total cost from €190.77 to €200.84 (see Figure 2). In *Situations 2* and *3*, costs were most sensitive to the duration of the SAQ analysis and the research technician unit cost, respectively. If we extrapolate these results to the French national level, with an incidence of 39,495 lung cancers per year [36], assuming the same distribution of patients in the three situations, the average cost would be €2,474,434, i.e., €62.65 per patient.

The mean duration of hospitalization was 40.9 days (SD 49.5; 95% CI 19.5–62.3) and the mean number of day-hospitalizations was 17.0 (SD 2.9; 95% CI 11.3–22.6). The mean annual cost of hospitalizations for occupational lung cancer was €14,778 (SD €6.312; 95% CI €11.9–17.6). The global cost for the 19 occupational lung cancer patients was €280,782. In the French context, with an incidence of 39,495 patients and a proportion of occupational lung cancers of between 9% and 18%, the annual hospitalization cost for occupational lung cancer was €52,529,140 and €105,058,280.

## 4. Discussion

Our study evaluated a systematic approach to identify occupational exposure in lung cancer patients attending a comprehensive cancer center in France to improve the reporting of occupational lung cancers. A medical compensation certificate was delivered to 18% of patients in our population and overall 8% have received compensation, to date. Our results are consistent with published estimates of the attributable fraction [15,18,19] and showed the capacity of our approach to improve compensation in lung cancer patients (i.e., 2.3% of compensated lung cancer in France in 2014 [20]). Despite its interdiction in France since 1997, asbestos is still the predominant carcinogen identified in occupational lung cancers as the incidence is estimated between 1328 and 3709 cases (i.e., 3.3 to 9.4% of lung cancers) per year and 2.1% were compensated in 2014. In our cohort, compensated occupational lung cancers related to asbestos represented 3.6% of the whole cohort and 6.8% of responders. Our results confirmed the recent estimates that more than half of occupational lung cancers are attributable to asbestos [37,38].

Near half of our responders were socially deprived, which is consistent with the literature, that shows lung cancer patients have a lower socio-economic status than the general population [12,39]. In our population, the level of deprivation may have been even higher since deprivation could be a barrier to complete the SAQ.

Our results showed that the SAQ could identify patients potentially exposed to occupational carcinogens. Since there are no specific diagnostic features for occupational-related lung cancers coupled with the confusing role of tobacco, physicians’ limited time to collect occupational history, a systematic SAQ to be completed by the patient at home could be an efficient approach to improve the screening of occupational exposures and thus overcome some of the barriers previously identified [22,24,28]. In our study, 45% of the SAQs were returned after a reminder, underlying the need of patients to be helped to recall their work history, especially deprived patients. Screening using a SAQ enables patients to be selected for referral for an occupational consultation rather than referring all patients systematically which is a more optimal use of time and resources.

The French National Health Insurance compensation procedure is often longer than the life expectancy of patients with lung cancer. The procedure took generally less than three months for patients who only had a few jobs and/or those reporting substantial asbestos exposure; it was longer for patients who had numerous jobs or who have been exposed to multiple carcinogens. For these latter patients, additional information was frequently requested, sometimes in an unclear manner. Due to this administrative burden, some patients abandoned their compensation claim. The administrative procedures were particularly complex for patients who had changed from being an employee to being self-employed which, in France, also leads to a change in their health insurance affiliation, which could be another barrier to compensation claims [24]. Besides, as the assessment is only based on a national list, there are some discrepancies between the occupational exposures from IARC’s group 1 carcinogens and what is possible to report and claim for compensation under the French system.

We estimated that the mean cost for the generalization of the systematic screening of occupational exposure in patients with lung cancer was €62.65 per patient. Our estimated annual costs of hospitalization for occupational lung cancer are consistent with those reported previously for France [40]. Increased transparency of the economic burden of occupational cancers for the healthcare system and society is important for the prioritization of occupational health policies and cancer prevention. The cost process could be transferable to other countries.

The low response rate to the SAQ (53%) is a limitation in our study. From their administrative records, at least 25% of the non-responders had held jobs potentially associated with exposure to occupational carcinogens. Some of the barriers we identified were similar to those previously reported [24,41]. Other barriers were also identified, such as reading comprehension, which can be associated with social deprivation, and which meant that some patients did not understand the purpose of the SAQ. In addition, although we tried to find an appointment when the patient was at the hospital, when possible, the consultation non-attendance rate was high. Eight patients (23%) with a medical certificate also abandoned the process as they did not claim for compensation. These results show the importance of accompanying the patients during the whole process, including the compensation claim. Most of the patients who came to the consultation had never heard about occupational diseases and the possibility to claim for compensation, which is consistent with the lack of information about occupational diseases and the necessity to improve the current process for patients’ rights.

More patients with a newly diagnosed lung cancer returned the SAQ than patients in progression, which may be explained by the fact that patients with progressive disease are generally tired due to cancer treatments. Sending the SAQ early after lung cancer diagnosis might thus improve the response rate.

## 5. Conclusions

Our study confirms the frequency of occupational exposures among patients with lung cancer, social deprivation in this population and the necessity to accompany patients during the compensation process. The information and education needs of exposed workers, patients, and healthcare professionals to improve the identification, reporting and compensation of occupational-related lung cancers must be a public health priority. 

As the feasibility of our systematic screening process and its capacity to improve compensation in lung cancer patients have been demonstrated, it could be implemented in other hospitals and for other pathologies. The CLB has already started to test this process in patients with lymphoma, in collaboration with another hospital in Lyon (Centre Hospitalier Lyon Sud). In parallel, our screening process will be implemented in six comprehensive cancer centers in patients with lung cancer.

## Figures and Tables

**Table 1:** Summary of patients’ characteristics overall and by smoking status.

	Overall	Current or ex-Smoker	Non-Smokers	*p* Value
	*N* (%)	*N* (%)	*N* (%)	
Total	439 ^1^ (100)	367 (84)	72 (16)	
Gender				
Male	278 (63)	265 (72)	13 (18)	<0.001
Female	161 (37)	102 (28)	59 (82)	
Mean age at diagnosis (SD) ^2^	63.0 (10.8)	62.5 (10.6)	65.7 (11.5)	0.03
Histologic type				0.007
Non-small cell lung carcinoma	399 (91)	331 (90)	68 (94)	
Adenocarcinoma	294 (67)	232 (63)	62 (86)	
Squamous cell carcinoma	77 (18)	72 (20)	5 (7)	
Undifferentiated large-cell carcinoma	15 (3)	14 (4)	1 (1)	
NSCLC non-defined	13 (3)	13 (3)	0 (0)	
Neuroendocrine	40 (9)	36 (10)	4 (6)	
Small cell lung carcinoma	30 (7)	28 (8)	2 (3)	
Large-cell carcinoma	6 (1)	5 (1)	1 (2)	
Carcinoid	4 (1)	3 (1)	1 (2)	
Stage ^3^				0.11
I	71 (16)	61 (17)	10 (14)	
II	38 (9)	33 (9)	5 (7)	
III	87 (20)	79 (21)	8 (11)	
IV	242 (55)	194 (53)	48 (67)	
Missing data	1 (0)		1 (1)	

^1^ Smoking status was missing for 1 patient; ^2^ SD = standard deviation (for quantitative data); ^3^ TNM Classification of malignant tumors, 8th Edition.

**Table 2:** Summary of the characteristics for self-administered questionnaire responders and non-responders.

	Responders	Non-Responders	*p* Value
	*N* (%)	*N* (%)	
Gender			
Men	154 (80)	125 (61)	0.31
Women	66 (34)	81 (39)	
Mean age at diagnosis (SD) ^1^	63.6 (10.0)	62.4 (11.7)	0.24
Tobacco			
Non-smokers	41 (17)	31 (15)	0.58
Smokers/Former smokers	193 (83)	174 (85)	
Pack-years (mean)	39.5	38.0	0.51
Status			
Newly diagnosed	141 (60)	135 (66)	0.29
Progression	93 (40)	71 (34)	
Stage			
Localized lung cancer	111 (47)	86 (42)	0.27
Metastatic lung cancer	123 (53)	120 (58)	

^1^ SD = standard deviation (for quantitative data).

**Table 3:** Number of Self-Administered Questionnaires (SAQ) returned and reasons for non-return.

	Patients
	*N* (%)
Total number of SAQ sent	440 (100)
Returned by patient without reminder	129 (29)
Returned after phone call reminder	105 (24)
By patient	85 (19)
Completed during the call	18 (4)
Completed by a beneficiary (patient deceased)	2 (0)
SAQ non-returned (reasons given during the phone call reminder)	174 (40)
Patient not concerned	84 (19)
Patient should have returned the SAQ but did not	32 (7)
Patient deceased	22 (5)
Fatigue	19 (4)
SAQ completed but never received	10 (2)
Patient did not want to complete the SAQ	9 (2)
Problems with French language	4 (1)
Patients already compensated	1 (0)
Patients could not be reached (after 3 attempts)	32 (7)

**Table 4:** Occupations and exposures of compensated patients.

Occupations	Exposure	Imputability
Electricians in construction industry (*N* = 3)	Asbestos	High
	Silica	Low
	Welding fumes and gases	Low
	Asbestos	Moderate
	Asbestos	Moderate
Boilermaker/sheet metal worker (*N* = 1)	Asbestos	High
Welders and oxy cutters (*N* = 2)	Asbestos	High
	Welding fumes and gases	High
	Asbestos	Moderate
	Welding fumes and gases	Low
Painters in construction industry, wallpaper installers (*N* = 2)	Asbestos	High
	Paint, varnish, lacquer, mastic	High
	Welding fumes and gases	Low
	Asbestos	Moderate
	Silica	Low
	Paint	Low
Driver of incinerators and water treatment process (*N* = 1)	Asbestos	Moderate
	Petroleum solvent	Low
Nurse (*N* = 1)	Ionizing radiation	Low
Bricklayers (*N* = 2)	Asbestos	High
	Crystalline silica	Moderate
	Asbestos	High
	Crystalline silica	Low
Automobile mechanic (*N* = 2)	Asbestos	Moderate
	Paint, varnish, lacquer, mastic	Moderate
	Welding fumes and gases	Low
Agricultural and industrial equipment mechanic (*N* = 1)	Asbestos	Moderate
	Welding fumes and gases	Low
	Exhaust diesel	Low
Roofing and zinc cladding worker (*N* = 1)	Asbestos	High
	Asphalt	Moderate
Driver of steam engines and boilers (*N* = 1)	Asbestos	High
	Burning soot	Low
Electrical and electronic engine fitter (*N* = 1)	Asbestos	Moderate
Driver of energy production equipment (*N* = 1)	Ionizing radiation	High

**Table 5:** Mean costs associated with systematic screening for occupational exposure in patients with lung cancer (microcosting approach).

Items	*Situation 1* (*N* = 77)		*Situation 2* (*N* = 82)		*Situation 3* (*N* = 142)	
	Mean Cost per Patient (€)	Percentage of Total	Mean Cost per Patient (€)	Percentage of Total	Mean Cost per Patient (€)	Percentage of Total
Personnel costs	147.22	77.1	16.32	66.6	7.79	56.9
Information system cost	2.99	1.6	2.99	12.2	2.99	21.8
Direct costs	150.21	78.7	19.31	78.8	10.78	78.7
Structure costs	40.56	21.3	5.21	21.2	2.91	21.3
Total cost	190.77	100.0	24.52	100.0	13.69	100.0

*Situation 1*: SAQ returned by the patient and consultation required based on the information provided by the patient in the SAQ. *Situation 2*: SAQ returned by the patient, no consultation required. *Situation 3*: SAQ not returned by the patient after the phone-reminder.

## Supplementary Materials

The following are available online at www.mdpi.com/1660-4601/15/1/65/s1, S1: Cost assessment of the systematic screening for occupational exposures: method, formulas and results.
