# Supplementary material for: Systematic Screening for Occupational Exposures in Lung Cancer Patients: A Prospective French Cohort
Source: Int J Environ Res Public Health. 2018 Jan 4;15(1):65. doi: 10.3390/ijerph15010065 (PMC5800164; doi:10.3390/ijerph15010065)
Supplement: Supplementary file 1 [file ijerph-15-00065-s001.pdf]

S1: cost assessment of the systematic screening for occupational exposures: method, formulas and results

1. Personnel costs

Measurements of the time required from staff for each specific task were assessed for each patient by using manual chronometers. For each category of staff (i.e. physician, technician, social worker, medical assistant), times were then multiplied by the unit wage costs.

Formaly, the cost C_{p_i} for a personnel resource p_i with

$$i \in \{\text{Physician, Clinical research assistant, Social worker, Medical assistant}\}$$

is expressed as:

$$C_{p_i} = UWC_{p_i} \times t_a = \frac{AWC_{p_i}}{AWT_{p_i}} \qquad (A1)$$

with UWC $_{p_i}$ the unit wage cost (all taxes included) of the personnel p_i , AWC $_{p_i}$ the annual wage cost of of the personnel p_i , AWT $_{p_i}$ the annual working time of the personnel p_i and t_a the duration of activity a.

2. Health information system cost

The health information system (HIS) cost per consultation including coding, software, maintenance and hardware was calculated by the accounting department with the courtesy of the Information System board.

Formaly,

$$HIS\ costs = costs\ of\ coding + software + maintenance + hardware \qquad (A2)$$

With

$$C_{coding} = \frac{Payroll\ coding\ team}{Nb\ of\ consultations\ in\ 2014} \times K_1$$

$$C_{software} = \frac{Annuity\ of\ software\ amortization}{Nb\ of\ consultations\ in\ 2014} \times K_2$$

$$C_{maintenance} = \frac{Maintenance\ cost}{Nb\ of\ consultations\ in\ 2014} \times K_3$$

$$C_{hardware} = \left(\frac{(\sum Computer\ invest.)/Amort.duration}{Nb\ of\ computers}\right)$$

And

$$K_1 = \frac{\sum Coding\ days\ for\ HIS\ (consultation)}{\sum Total\ coding\ days\ for\ HIS}$$

$$K_2 = \frac{\sum Software\ investment\ (consultation)}{\sum Total\ software\ investment}$$

$$K_3 = \frac{\sum Maintenance\ investment\ (consultation)}{\sum Total\ maintenance\ investment}$$

3. Costs of structure

Costs of structure (e.g. the costs of logistics and general management) were determined to be 27% of the other costs based on the ENCC (National Study on Common Costs) data (http://www.atih.sante.fr/sites/default/files/public/content/70/atih_national_cost_studies.pdf).

4. Total cost

$CT = (\sum_{I=1}^{4} C_P + HIS\ cost) \times 1.27$ (A3)
